# Author Correction: Toll‐like receptor 9 protects non‐immune cells from stress by modulating mitochondrial ATP synthesis through the inhibition of SERCA2

**DOI:** 10.1038/s44319-024-00100-1

**Published:** 2024-03-01

**Authors:** Yasunori Shintani, Hannes CA Drexler, Hidetaka Kioka, Cesare MN Terracciano, Steven R Coppen, Hiromi Imamura, Masaharu Akao, Junichi Nakai, Ann P Wheeler, Shuichiro Higo, Hiroyuki Nakayama, Seiji Takashima, Kenta Yashiro, Ken Suzuki

**Affiliations:** 1 William Harvey Research Institute, Barts and The London School of Medicine and Dentistry, Queen Mary University of London, Charterhouse Square, London, EC1M 6BQ UK; 2 Department of Medical Biochemistry, Osaka University Graduate School of Medicine, Suita, Osaka, 565-0871 Japan; 3 Bioanalytical Mass Spectrometry, Max Planck Institute for Molecular Biomedicine, Röntgenstr. 20, 48149 Muenster, Germany; 4 Department of Cardiovascular Medicine, Osaka University Graduate School of Medicine, Suita, Osaka, 565-0871 Japan; 5 Laboratory of Myocardial Electrophysiology, Imperial Centre for Translational and Experimental Medicine, Imperial College London, National Heart & Lung Institute, Hammersmith Campus, Du Cane Road, London, W12 0NN UK; 6 The Hakubi Center & Graduate School of Biostudies, Kyoto University Science Frontier Laboratory building Room 305, Faculty of Medicine Campus, Kyoto, 606-8501 Japan; 7 Department of Cardiology, National Hospital Organization Kyoto Medical Center, Kyoto, 612-8555 Japan; 8 Saitama University Brain Science Institute, Saitama City, 338-8570 Japan; 9 Blizard Advanced Light Microscopy Facility, Blizard Institute, Barts and The London School of Medicine and Dentistry, Queen Mary University of London, London, E1 2AT UK; 10 Laboratory of Clinical Science and Biomedicine, Osaka University Graduate School of Pharmaceutical Sciences, Osaka, 565-0871 Japan

## Abstract

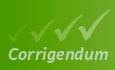

**Correction to:** *EMBO Reports* (2014) 15:438–445. 10.1002/embr.201337945 | Published online 7 March 2014

The authors contacted the Journal after a panel re-use was identified in the manuscript without proper citation.

**Figure 2B is retracted. Associated text is updated**.

**The following text has been updated**.

Page 2 of manuscript PDF.

Finally, immunofluorescence studies demonstrated that not only the overexpressed TLR9-HA-FLAG protein colocalized with SERCA2 **(panel 3A—in reference 6)** and with the ER/SR marker KDEL in cardiomyocytes (Fig 2B and Supplementary Fig S1C online), but also the endogenous TLR9 colocalized with SERCA2 (Fig 2C). Collectively from these data, we conclude that TLR9 interacts with SERCA2 at ER/SR in cardiomyocytes.

Author Comments:

It has recently been brought to our attention that we reused the panels in Figure 2B from our paper published earlier (Figure 3A, upper, 0 min. PNAS. 2013;110(13):5109-14. 10.1073/pnas.1219243110). We therefore remove the Figure 2B panels and revise the corresponding manuscript text.

This error does not affect the conclusions of the original paper. All authors agree to this correction and apologize for this error.

