# Supplementary material for: Toll-like receptor 9 protects non-immune cells from stress by modulating mitochondrial ATP synthesis through the inhibition of SERCA2
Source: EMBO Rep. 2014 Mar 7;15(4):438–45. doi: 10.1002/embr.201337945 (PMC3989675; doi:10.1002/embr.201337945)
Supplement: Supplementary file 1 — Supplementary Figure S1 (PDF 604 KB) [file 41586_2014_BFEMBR201337945_MOESM1_ESM.pdf]

Figure S1

A

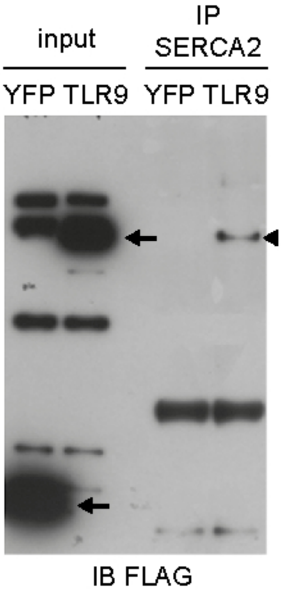

B

	Cardiomyocytes		Cardiac Fibroblasts	
Gene Names	TLR9 FL	YFP	TLR9 FL	YFP
Grp78	89		67	
Hspa8	22		43	
Tlr9	19		16	
Hspb1	12		9	
Atp5b	10		4	
Canx	9		9	
Cryab	8		16	3
Atp5a1	6		1	1
Rcn2	6		11	
Casq2	5			
Serca2	5			
Dnajb11	3		2	
Atp5o	2			
Ndufa4	2			
Tubb	2		8	
Ubc	2		1	1
Rps15a	1			
Rps16	1			
Rps24	1			
Rpl13	1		1	1
Rpl23	1			
Rpl28	1			
ABCA1	1			
Atp5c1	1			
Calr	1		1	
Dnaja2	1		1	1
Sdhb	1			
Tmem33	1		5	
Fau	1		1	2

C

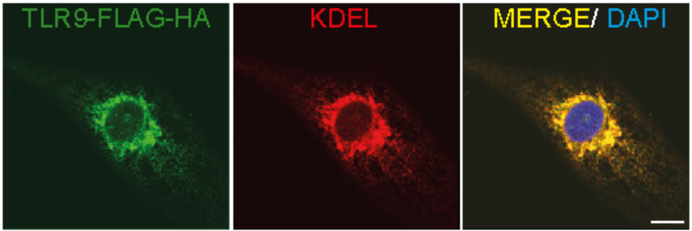

Figure S1 SERCA2 is a binding protein of TLR9

(A) Reciprocal immunoprecipitation with SERCA2 antibody demonstrated that SERCA2 associated with TLR9, but not with YFP. YFP-HA-FLAG or TLR9-HA-FLAG were transfected in cardiomyocytes, lysed and immunoprecipitated with SERCA2 antibody, then immunoblotted with FLAG. Arrows; overexpressed YFP or TLR9 in input. Arrowhead; co-immunoprecipitated TLR9. (B) The list of identified proteins that associated with overexpressed TLR9-HA-FLAG or YFP-HA-FLAG by LCMSMS in cardiomyocytes or cardiac fibroblasts. Values represent the number of identified peptides. The proteins were selected by the following criteria; TLR9FL > 1 and YFP = 0 in cardiomyocytes. SERCA2 was identified in cardiomyocytes immunoprecipitates, but not in fibroblasts, while most of heat shock proteins or abundant proteins are found in immunoprecipitate from both cell types. Notably, other abundant Ca^{2+} pump proteins in cardiomyocytes, such as ryanodine receptor (RyR) or inositol 1,4,5-triphosphate receptor (IP3R), were not detected. (C) The overexpressed TLR9 localized at the ER/SR. TLR9-HA-FLAG was expressed in cardiomyocytes and labeled with anti-HA antibody (green) in addition to KDEL (red) antibody. Scale bar indicates 10 μm.
