# Supplementary material for: Toll-like receptor 9 protects non-immune cells from stress by modulating mitochondrial ATP synthesis through the inhibition of SERCA2
Source: EMBO Rep. 2014 Mar 7;15(4):438–45. doi: 10.1002/embr.201337945 (PMC3989675; doi:10.1002/embr.201337945)
Supplement: Supplementary file 2 — Supplementary Figure S2 (PDF 380 KB) [file 41586_2014_BFEMBR201337945_MOESM2_ESM.pdf]

Figure S2

A

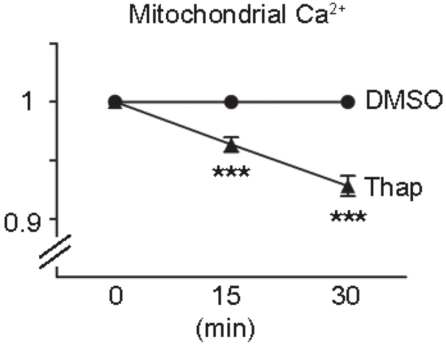

B

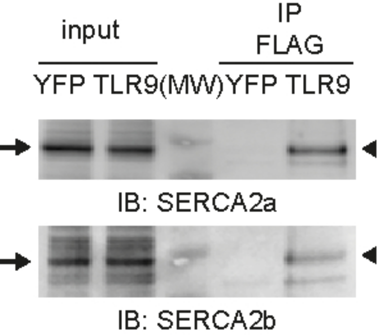

C

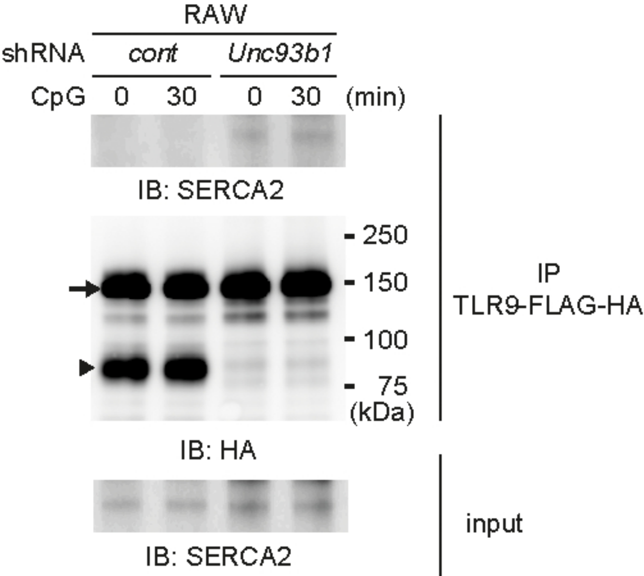

Figure S2

(A) Measurement of [Ca2+]m in GCaMP2-mt transfected cardiomyocytes after administration of control vehicle (DMSO; n=19, circle) or thapsigargin (500 ng/ml, n=22, triangle). [Ca2+]m significantly dropped at 15 and 30 min after thapsigargin administration. Error bars indicate SEM. ***p<0.001 compared with the control. (B) TLR9 associated with both SERCA2 isoforms. YFP-HA-FLAG or TLR9-HA-FLAG encoded adenoviruses were transfected in rat cardiomyocytes, lysed and immunoprecipitated with FLAG M2 agarose, then detected by the isoform specific SERCA2 antibodies. Arrows; SERCA2a or b in 1% input of the total lysates, Arrowhead; co-immunoprecipitated SERCA2a or b, respectively. (C) The interaction between TLR9 and SERCA2 was found in *Unc93b1* knocked-down RAW264.7 cells, while no such interaction in control cells. TLR9 were stably transfected in RAW264.7 cells, in which shRNA for *Unc93b1* or *control* had been already transfected. The cell lysate were immunoprecipitated with anti-FLAG antibody at 0 and 30 min after CpG stimulation, immunoblotted with SERCA2 antibody. 1% of whole cell lysates were used as input. The molecular weight of overexpressed TLR9 matched with the full-length form (arrow) and the cleaved form (arrowhead) in the control cells, while only full-length form was found in *Unc93b1* knocked-down RAW264.7 cells.
