# Supplementary material for: Toll-like receptor 9 protects non-immune cells from stress by modulating mitochondrial ATP synthesis through the inhibition of SERCA2
Source: EMBO Rep. 2014 Mar 7;15(4):438–45. doi: 10.1002/embr.201337945 (PMC3989675; doi:10.1002/embr.201337945)
Supplement: Supplementary file 3 — Supplementary Figure S3 (PDF 372 KB) [file 41586_2014_BFEMBR201337945_MOESM3_ESM.pdf]

Figure S3

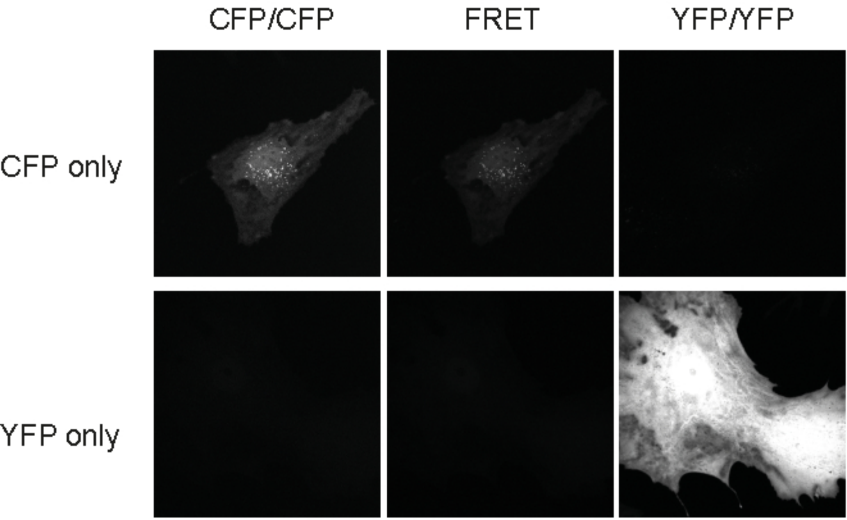

Figure S3. Bleed-through of fluorescence emission into other fluorescence channels.
Fluorescence images in CFP/CFP, FRET (CFP/YFP) and YFP/YFP channels of HEK293 cells that were transfected with CFP only expressing construct (top row) or YFP only expressing construct (bottom row). TLR9 associated with SERCA2 in Unc93b1 knocked down RAW264.7 cells, but not in control RAW264.7 cells.
