# Supplementary material for: Toll-like receptor 9 protects non-immune cells from stress by modulating mitochondrial ATP synthesis through the inhibition of SERCA2
Source: EMBO Rep. 2014 Mar 7;15(4):438–45. doi: 10.1002/embr.201337945 (PMC3989675; doi:10.1002/embr.201337945)
Supplement: Supplementary file 4 — Supplementary Methods (PDF 128 KB) [file 41586_2014_BFEMBR201337945_MOESM4_ESM.pdf]

Supplementary Methods

Mice and reagents

TLR9$^{-/-}$ mice were generated in Professor Akira's laboratory and purchased from Oriental Bioservice with C57BL6 background (Kyoto, Japan). C57BL6 wild-type mice were obtained from Charles River, UK. CpG-ODN 1668 (CpG type B) and were purchased from Source Bioscience. Oligomycin A and thapsigargin were purchased from Sigma.

Plasmids and viral vector construction

Mouse TLR9-myc cDNA was a kind gift from Dr Brinkmann. C-terminal myc was replaced with HA-FLAG tag by a PCR-based method. The amplified fragment was inserted to pENTR1A (Invitrogen) and the sequence was verified. The subcloned cDNA was transferred to pAd/CMV/V5-DEST (Invitrogen) using Gateway system (Invitrogen). Adenovirus was generated using ViraPower Adenoviral Expression System (Invitrogen) as described by the manufacturer, following purification using Vivapure AdenoPACK (Satorius Stedim Biotech).

Antibodies

Isoform-specific antibodies for SERCA2a and b antibodies were kind gift from Dr. Wuytack. Antibodies for TLR9 (GeneTex; 5G5), SERCA2 (Abcam; ab3625 [rabbit] for immunofluorescence and immunoprecipitation, Santa Cruz; sc-8095 for Western blotting), phopsho-AMPK (Cell signalling; #2535), phospho-ACC (Cell signalling; #3661), tubulin (Sigma; B-5-1-2), FLAG (Sigma; F1804 for immunofluorescence and Western blotting), HA (Roche; 3F10 for immunofluorescence and Western blotting), phospho-Ser16 phopholamban (Badrilla), phospho-Thr17 phopholamban (Santa Cruz; sc-17024-R) and KDEL (Enzo; 10C3) were purchased. Anti-HA (clone 12CA5) and anti-Flag (M2) affinity matrices (A2220) were purchased from Roche and Sigma, respectively.

Cell culture

Primary cultures of neonatal rat cardiomyocytes were prepared from 2-3 days old Wistar rats. Harvested hearts were incubated in 0.125% trypsin/EDTA (Sigma) at 4°C overnight, and then digested with collagenase type II (Worthington). The cardiomyocyte fraction was collected after differential plating for 70 min at 37°C, counted and seeded onto gelatin-coated dishes, or fibronectin coated glass-bottom dishes. For mouse neonatal cardiomyocytes, 0.025% trypsin/EDTA was used. Cardiomyocytes were cultured in DMEM (Sigma) supplemented with 5% FBS. One day after isolation, cardiomyocytes were treated with mitomycin C (10 μg/ml; Sigma) for 2.5 hrs to inhibit growth of other cell types contaminating the culture. Cells were normally used for experiments 4-6 days after isolation. The medium was changed to DMEM

containing 2% FBS for 30 min prior to CpG-ODN administration. For Ca^{2+} transient and mitochondrial Ca^{2+} experiment, medium were supplemented with 3.6 mM Ca^{2+}. RAW264.7 cells were obtained from ECACC and maintained in DMEM supplemented with 10% FBS. For *Unc93b1* knocked-down cells, we prepared them as previously described [1].

LC-MSMS

Bands of interest were cut from the silverstained gel and diced into small cubes of approximately $1mm^3$ in size. The gel pieces were destained for 10 min in Farmer´s Reducer (15 mM potassium ferricyanide, 50 mM sodium thiosulfate) prior to in-gel digestion with trypsin [2]. Extracted peptides were desalted using C18 reversed phase material and stored at 4°C until analyzed by LC-MS/MS. To this end, an EasyLC nanoflow system (Proxeon) was employed that was online-coupled *via* in-house packed fused silica capillary column emitters (length 15 cm; ID 75 µm; resin ReproSil-Pur C18-AQ, 3 µm) and a nanoelectrospray ion source (Proxeon) to an LTQ Orbitrap Velos mass spectrometer (Thermo Scientific). Peptide separation was accomplished by applying a multi-segment gradient (5-7% buffer B in 2 min; 7-32% B in 90min; 32-60% B in 10 min; 60-80% B in 6 min; 80-98% B in 1 min; buffer B (80% acetonitril, 0.5% acetic acid)). The mass spectrometer was operated in the positive ion mode (source voltage 2.2 kV), automatically switching in a data-dependent fashion between survey scans in the mass range of m/z 300_1650 and MS/MS acquisition. Collision induced MS/MS spectra from the 15 most intense ion peaks in the MS were collected (Target Value of the Orbitrap survey scan: 1000000; resolution R = 60000; Lockmass set to 445.120025). To increase proteome coverage dynamic exclusion was enabled (repeat count 1; repeat duration 30s; exclusion list size 500; exclusion duration 120s). Raw data files were then processed by MaxQuant software (v 1.0.13.13) in conjunction with Mascot database searches (version 2.2). Data were searched against the International Protein Index sequence database (rat IPI, version 3.60) concatenated with reversed sequence versions of all entries. The parameter settings were: Trypsin as digesting enzyme, a minimum length of 6 amino acids, a maximum of 2 missed cleavages, carbamidomethylation at cysteine residues set as fixed and oxidation at methionine residues as well as acetylation at the protein N-termini as variable modifications. The maximum allowed mass deviation was 7 ppm for MS and 0.5 Da for MS/MS scans. Protein groups were regarded as being unequivocally identified with a false discovery rate (FDR) set to 1% for all protein as well as peptide identifications when there were at least 2 matching peptides, one of which being unique to the protein group.

Co-immunoprecipitation with intracellular crosslinking

Co-immunoprecipitation with intracellular crosslinking was performed as previously reported with slight modification [3]. $5x10^6$ neonatal cardiomyocytes were plated in 100 mm dish. Cells

were treated with or without CpG-ODN for 20 min. Cells were then incubated with 1.5 mM DSP (Pierce) in HBSS for 2 h at 4°C and cross-linking was terminated with 20 mM Tris for 30 min at 4°C. Cell lysate was extracted in RIPA buffer (25 mM Tris, pH 7.6, 0.15 M NaCl, 1% sodium deoxycholate, 1% NP40) containing protease inhibitor tablet (Complete, Roche). The lysate was precleared by incubation with 2 μg of normal rabbit IgG and 20 μl of Dynabeads proteinG beads (Life technologies) for 1 h at 4°C. After preclearing, the lysates were incubated with 2 μg of rabbit anti-SERCA2 antibody (Abcam; ab3625) or normal rabbit IgG for 16 h at 4°C. The beads were recovered by centrifugation and washed five times with lysis buffer. Finally, reversal of crosslinking and elution of the immunoprecipitated proteins were performed in sample buffer containing 50 mM DTT for 5 min at 98 °C.

FRET-based measurement of SR/ER Ca^{2+}

Cardiomyocytes were infected with adenovirus encoding FRET-based Ca^{2+} indicators D1ER to measure changes in SR/ER Ca^{2+} concentrations [4,5]. Wide-field observations of the cells were performed on an Olympus IX-81 inverted fluorescence microscope (Olympus) using a PLAPO 60X, 1.35 NA, oil immersion objective lens (Olympus). Fluorescence emission from ATeam was imaged by using a dual cooled charge-coupled device (CCD) camera (ORCA-D2; Hamamatsu Photonics) with a dichroic mirror 510 nm and two emission filters (483 nm/32 nm for CFP and 542 nm/27 nm for YFP; A11400-03, Hamamatsu Photonics). Cells were illuminated using CoolLED *p*E-1 excitation system (CoolLED) with a wavelength of 425 nm. Cells were maintained on a microscope at 37°C using a stage-top incubator (Tokai Hit). Image analysis was performed using MetaMorph (Molecular Devices). The FRET/CFP emission ratio was calculated by dividing pixel-by pixel an FRET image with a CFP image after background subtraction.

SERCA2 ATPase activity assay

SERCA2 ATPase activity was measured with an enzyme-coupled spectrophotometric assay in which hydrolysis of ATP is coupled to the oxidation of NADH, as previously described [6-8]. Microsome fraction was prepared using the method previously described [9]. 5μg of microsome fraction was incubated in buffer containg MOPS 21 mM, NaN_3 4.9 mM, EGTA 0.06 mM, KCl 100 mM, glycerophosphate 1 mM, $MgCl_2$ 3 mM, NADH 0.1 mM, phophoenolpyruvate 1 mM, pyruvate kinase 8.4 units, lactate dehydrogenase 12 units. The reaction was started with the addition of ATP (1 mM) and performed at 30°C. The hydrolysis of ATP by SERCA2 activity is considered as an equivalent to NADH generation and the reaction was monitored photometrically with a spectrophotometer. SERCA2 activity is calculated from the change of absorption at 340 nm divided by the extinction coefficient of NADH (6.22 cm^2/μmol) per mg protein x min. Confirmation of Ca^{2+} dependency and basal activity was done with Ca^{2+}-free

buffer in the presence of EGTA 4 mM.

Ca^{2+} transient and Ca^{2+} content analysis

Rat adult cardiomyocytes isolation and Ca^{2+} transients analysis were performed as previously described [10]. Ca^{2+} transients were analyzed in rat neonatal cardiomyocytes plated in 35 mm glass-bottom dish. Cardiomyocytes were loaded with the fluorescent indicator Fluo-4 AM (10 μM, Molecular Probes) at 5% CO2 at 37°C for 20 min. Then, cells were treated with or without CpG-ODN (type B; 3 μM) for 30 min before being mounted on a confocal microscope (Zeiss LSM 510) for analysis. Cells were stimulated by field stimulator with 0.5 Hz pacing. We allowed 5 min for cells to adapt to pacing. Fluorescence measured across cells using line scans. In off-line analysis, we selected 2 representative transients for each cell, then measured parameters, which were averaged. We collected the data (control; *n*=50 and CpG-ODN; *n*=51 cells) for each condition from 3 independent preparations, and then subjected them into statistical analysis. To measure caffeine-induced Fluo-4 transients as a measure of Ca^{2+} content in the ER/SR, field stimulation was stopped, and caffeine was rapidly applied using a solenoid switcher device. Immediately before the application of caffeine, the superfusate was switched to a Na-free/ Ca^{2+}-free solution (140 mM LiCl, 10 mM glucose, 10 mM HEPES, 0.75 mM EGTA, 1 mM $MgCl_2$, and 6 mM KOH, pH 7.4) to prevent Ca^{2+} extrusion by the Na/ Ca^{2+} exchanger. This was followed by an application of 20 mM caffeine dissolved in the same Na-free/ Ca^{2+}-free solution. The amplitude of the caffeine-induced transient was taken as a measure of Ca^{2+} content.

Mitochondrial Ca^{2+} measurement

Cardiomyocytes were transfected with G-CaMP2-mt adenovirus [11] 2 days before analysis. Transfected cardiomyocytes on 35-mm glass-bottom dishes were maintained on a microscope at 37°C with a continuous supply of a 95% air and 5% carbon dioxide mixture by using a stage-top incubator (Tokai Hit). Time-lapse images were obtained using Olympus IX81 inverted microscope at 1-min intervals with x60 oil-immersion lens (APO, NA 1.35, Olympus). Filters used (Semrock) were as follows; excitation filter (482/18-25), an FF495-Di02-25x36 dichroic mirror, and emission filter (531/22-25). Cells were illuminated using a 100W mercury lamp through 12.5% and 25% neutral density filters and exposure time was 300 msec. Image analysis was performed using MetaMorph (Molecular Devices). An average intensity/pixel from a whole cell was calculated. The mean value between -3 and 0 min was used as baseline, against which the data from each time point was normalized. The results are representative of at least three independent experiments.

FRET-based measurement of mitochondrial matrix ATP

Mit-ATeam [12,13] was subcloned into a pENTR1A vector, and the adenovirus was generated as described in the above section. Cardiomyocytes on glass-bottom dishes (Iwaki) were transfected with adenoviral mit-ATeam, and imaged 48 hrs after transfection. Time-lapse images were obtained with LSM510 inverted confocal microscope (Zeiss) with 458 nm Argon laser excitation. Corrected FRET (cFRET) was calculated using fully specified bleed through correction method. Under our experimental conditions (Supplementary Fig S3 online), donor in acceptor channel, acceptor in donor channel, donor in FRET channel and acceptor in FRET channel were 0.01031, 0.01787, 0.43003 and 0.0159 respectively: cFRET= FRET - 0.01959 x (YFP – 0.01031 x CFP) - 0.43003 x (CFP – 0.01787 x YFP). Image analysis was performed using MetaMorph (Molecular Devices). We recorded 1 to 2 cells per an experiment and performed at least 3 conditions (control, CpG-ODN, Oligomycin) per day.

RNAi

To knock down *SERCA2*, cardiomyocytes were transfected with of siRNAs (5 nM) using Lipofectamine RNAi MAX (Invitrogen) 4-6 hours after isolation; *SERCA2-1* (sense: cgcuagaguuuucacgugaTT; antisense: ucacgugaaaacucuagcgTG), *SERCA2-2* (sense: gccugcaacucggucauaaTT; antisense: uuaugaccgaguugcaggcGT) (Ambion). As a negative control, siControl (Ambion) was used.

Confocal microscopy

Cardiomyocytes were plated onto laminin or fibronectin coated 8 well chamber slides (Nunc) or glass-bottom dishes (IWAKI). Cells were then stimulated with or without CpG-ODN, washed with PBS and fixed with 4% PFA at room temperature for 10 min. After washing with PBS, cells were permeabilized with 0.1% TritonX100 and blocked with 5% goat serum or BSA. Primary antibodies were then applied and incubated at 4°C overnight. After labelling with Alexa 488 or 555-conjugated secondary antibody (Invitrogen), images were obtained using LSM 710 confocal microscopy (Zeiss) with 1 μm thickness using x63 objective lens. At least 20 cells were examined per condition and representative images were chosen.

Supplementary references

1. Shintani Y, Kapoor A, Kaneko M, Smolenski RT, D'Acquisto F, Coppen SR, Harada-Shoji N, Lee HJ, Thiemermann C, Takashima S, et al. (2013) TLR9 mediates cellular protection by modulating energy metabolism in cardiomyocytes and neurons. *Proc Natl Acad Sci USA* **110**: 5109–5114.
2. Shevchenko A, Tomas H, Havlis J, Olsen JV, Mann M (2006) In-gel digestion for mass spectrometric characterization of proteins and proteomes. *Nat Protoc* **1**: 2856–2860.
3. Zhang L, Rayner S, Katoku-Kikyo N, Romanova L, Kikyo N (2007) Successful co-immunoprecipitation of Oct4 and Nanog using cross-linking. *Biochem Biophys Res Commun* **361**: 611–614.
4. Palmer AE, Jin C, Reed JC, Tsien RY (2004) Bcl-2-mediated alterations in endoplasmic reticulum Ca2+ analyzed with an improved genetically encoded fluorescent sensor. *Proc Natl Acad Sci USA* **101**: 17404–17409.
5. Palmer AE, Tsien RY (2006) Measuring calcium signaling using genetically targetable fluorescent indicators. *Nat Protoc* **1**: 1057–1065.
6. Randriamboavonjy V, Pistrosch F, Bolck B, Schwinger RHG, Dixit M, Badenhoop K, Cohen RA, Busse R, Fleming I (2008) Platelet Sarcoplasmic Endoplasmic Reticulum Ca2+-ATPase and -Calpain Activity Are Altered in Type 2 Diabetes Mellitus and Restored by Rosiglitazone. *Circulation* **117**: 52–60.
7. Münch G (2002) Evidence for Calcineurin-mediated Regulation of SERCA 2a Activity in Human Myocardium. *J Mol Cell Cardiol* **34**: 321–334.
8. Chu A, Dixon MC, Saito A, Seiler S, Fleischer S (1988) Isolation of sarcoplasmic reticulum fractions referable to longitudinal tubules and junctional terminal cisternae from rabbit skeletal muscle. *Meth Enzymol* **157**: 36–46.
9. Ji Y, Loukianov E, Periasamy M (1999) Analysis of sarcoplasmic reticulum Ca2+ transport and Ca2+ ATPase enzymatic properties using mouse cardiac tissue homogenates. *Anal Biochem* **269**: 236–244.
10. Siedlecka U, Arora M, Kolettis T, Soppa GKR, Lee J, Stagg MA, Harding SE, Yacoub MH, Terracciano CMN (2008) Effects of clenbuterol on contractility and Ca2+ homeostasis of isolated rat ventricular myocytes. *AJP: Heart and Circulatory Physiology* **295**: H1917–H1926.
11. Iguchi M, Kato M, Nakai J, Takeda T, Matsumoto-Ida M, Kita T, Kimura T, Akao M (2011) Direct monitoring of mitochondrial calcium levels in cultured cardiac myocytes using a novel fluorescent indicator protein, GCaMP2-mt. *Int J Cardiol*.
12. Imamura H, Nhat KPH, Togawa H, Saito K, Iino R, Kato-Yamada Y, Nagai T, Noji H (2009) Visualization of ATP levels inside single living cells with fluorescence resonance

energy transfer-based genetically encoded indicators. *Proc Natl Acad Sci USA* **106**: 15651–15656.

13. Nakano M, Imamura H, Nagai T, Noji H (2011) Ca 2+Regulation of Mitochondrial ATP Synthesis Visualized at the Single Cell Level. *ACS Chem Biol* **6**: 709–715.
