# Supplementary figures and images for: Toll-like receptor 9 protects non-immune cells from stress by modulating mitochondrial ATP synthesis through the inhibition of SERCA2
Source: EMBO Rep. 2014 Mar 7;15(4):438–45. doi: 10.1002/embr.201337945 (PMC3989675; doi:10.1002/embr.201337945)

Fig. 1A

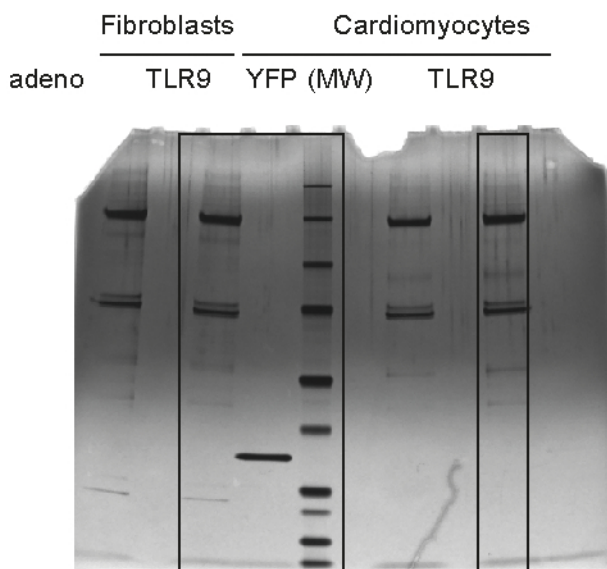

Fig. 1B

Cardiomyocytes  
CpG 0 15 min

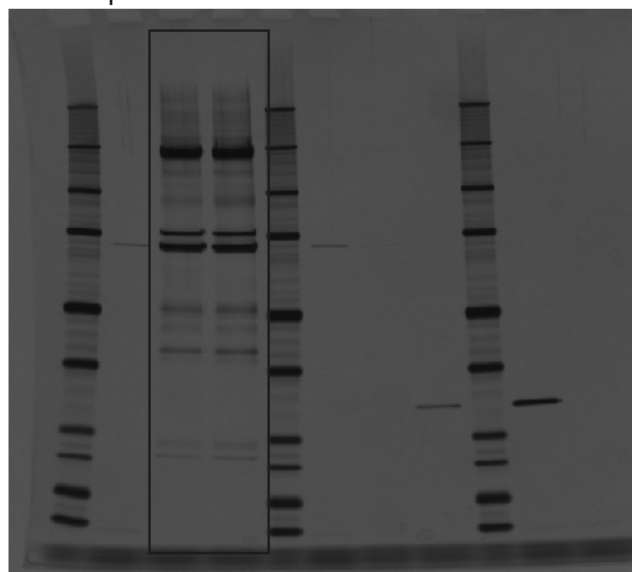

Supplement: Supplementary file 6 — Source Data for Figure 1 (PDF 688 KB) [file 41586_2014_BFEMBR201337945_MOESM6_ESM.pdf]

Fig. 2A

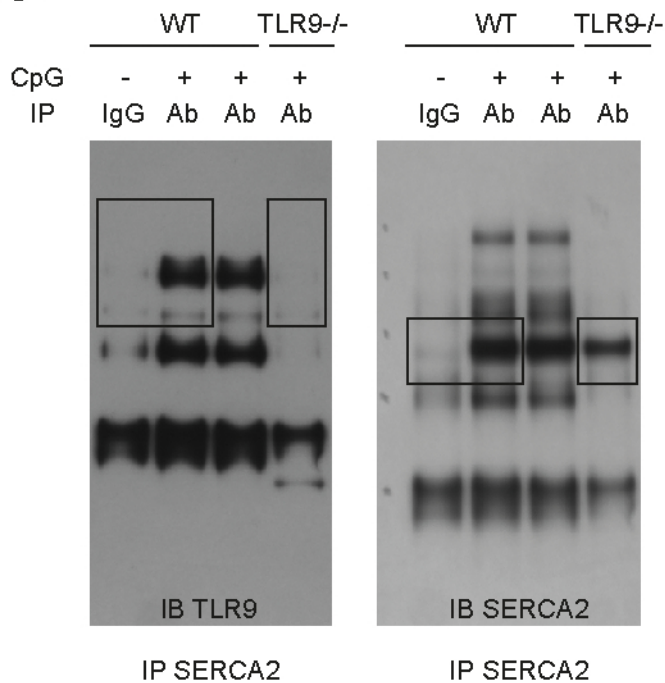

Supplement: Supplementary file 7 — Source Data for Figure 2 (PDF 388 KB) [file 41586_2014_BFEMBR201337945_MOESM7_ESM.pdf]

**Fig. 3A**

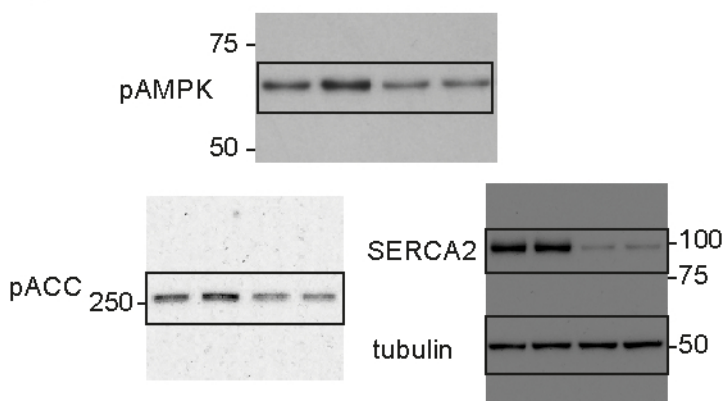

**Fig. 3H**

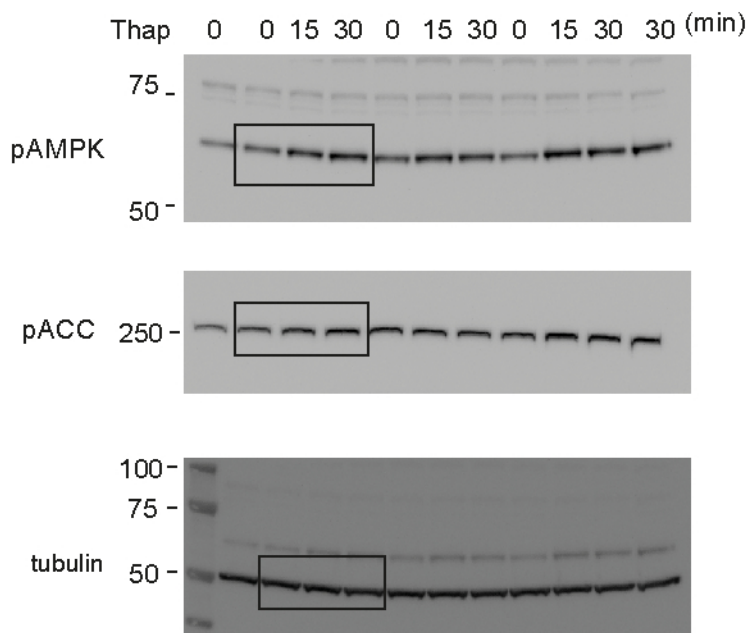

Supplement: Supplementary file 8 — Source Data for Figure 3 (PDF 540 KB) [file 41586_2014_BFEMBR201337945_MOESM8_ESM.pdf]
